# Short to Midterm Follow-Up of Periprosthetic Bone Mineral Density after Total Hip Arthroplasty with the Ribbed Anatomic Stem

**DOI:** 10.1155/2019/3085258

**Published:** 2019-06-27

**Authors:** Xiang-Dong Wu, Mian Tian, Yao He, Hong Chen, Yu Chen, Rahul Mishra, Wei Liu, Wei Huang

**Affiliations:** ^1^Department of Orthopaedic Surgery, The First Affiliated Hospital of Chongqing Medical University, Chongqing 400016, China; ^2^Department of Orthopaedic Surgery, Dianjiang People's Hospital, Chongqing 400060, China; ^3^Department of Orthopaedic Surgery, Banan People's Hospital of Chongqing, Chongqing 400320, China; ^4^Department of Orthopedics, Siddharth Hospital, Banke 21900, Nepal

## Abstract

**Background:**

Femoral bone remodeling around hip prosthesis after total hip arthroplasty (THA) is definite but unpredictable in time and place. This study aimed to investigate the implant-specific remodeling and periprosthetic bone mineral density (BMD) changes after implantation of the Ribbed anatomic cementless femoral stem.

**Methods:**

After power analysis, 41 patients who had undergone primary unilateral THA with the Ribbed anatomic cementless stem were included. BMD of the seven Gruen zones was measured by dual-energy X-ray absorptiometry, and the contact, fitness, and fixation of the femoral stem and proximal femur were analyzed by X-ray. Additional clinical outcome parameters were also recorded.

**Results:**

Compared with the contralateral unoperated side, significant reductions of BMD were detected in the distal zone (Gruen zone 4: 1.665±0.198* versus* 1.568±0.242 g/cm^2^,* P*=0.001) and middle distal zone (Gruen zone 5: 1.660±0.209* versus* 1.608±0.215 g/cm^2^,* P*=0.026) on the prosthetic side, but no significant differences in BMD were detected in other zones (Gruen zones 1, 2, 3, 6, and 7). Subgroups analyses indicated no significant correlation between periprosthetic BMD changes and clinical factors including primary disease and body mass index. Visible areas of bone ingrowth indicated solid fixation of the femoral stem and there was no case of loosening. Clinical and functional outcome scores were excellent with mean HHS of 93.13 points and mean WOMAC score of 5.20 points, and three patients described intermittent mild thigh pain at the final follow-up.

**Conclusions:**

For the Ribbed femoral stem, the periprosthetic BMD was well maintained in the proximal femur, while periprosthetic BMD was significantly reduced in the distal and middle distal zones of the femur. Further clinical investigations are required to examine the efficacy of the Ribbed stem, particularly with regard to long-term survival. This trial is registered with ChiCTR1800017750.

## 1. Introduction

Total hip arthroplasty (THA) is one of the most successful surgical procedures in the world, with universally high patient satisfaction above 90% [1–3]. The implant survival following THA is the most commonly investigated outcome, and recent studies suggested increased survivorship of implant in long-term follow-up [4–6]. However, with the projected exponential growth in primary THA and increased life expectancy [7–9], the volume of revision is expected to rise. Therefore, engendering clinical longevity of implants, achieving higher long-term survivorship of primary THA, and reducing revision rate should be given top priority.

The use of press-fit cementless femoral implants has increased substantially over the past decades and currently has become the mainstream in primary THA [10, 11]. Recent studies showed that the initial fixation and long-term stability of the components were determinants that affect implant survivorship [12, 13] and periprosthetic bone remodeling, which encompasses bone resorption as well as increase in bone density as a result of stress shielding or stress concentration, is associated with the long-term fixation of cementless implants [14–16]. Therefore, exploration of the femoral bone remodeling and periprosthetic bone mineral density (BMD) changes around hip prosthesis after implantation would help to evaluate the osseointegration [16, 17].

A number of cementless femoral stems are associated with excellent survivorship, but various types of designs are different in prosthesis geometry, means of obtaining primary fixation, proximal stress transfer, and stress shielding [18], thus induce implant-specific femoral remodeling pattern and BMD changes. Ribbed femoral stem is a representative of anatomic cementless prostheses and is widely used, but periprosthetic BMD changes after its insertion remain unclear. Therefore, this study aimed to investigate the femoral remodeling pattern and periprosthetic BMD changes in patients who undergo primary unilateral THA with the Ribbed femoral stem.

## 2. Materials and Methods

Ethical approval for the study was obtained through The First Affiliated Hospital of Chongqing Medical University.

### 2.1. Inclusion and Exclusion Criteria

The inclusion criteria were as follows: (1) adult patients who undergo primary unilateral THA with the Ribbed (Ribbed® Hip system, Waldemar Link®, Hamburg, Germany) anatomic cementless hydroxyapatite (HA) coated femoral stem in our department; (2) surgeries were performed by two senior surgeons using posterior-lateral approach under general anesthesia in laminar air flow operation room; (3) patients understood the scope of the study and agreed to participate at follow-up. Individuals diagnosed with intertrochanteric fractures or pathological fractures or diagnosed with any other diseases (bone tumor, hyperthyroidism, hypothyroidism, glucosteroid or corticosteroid use, etc.) that affect bone metabolism would be excluded from this study. In addition, patient who received a revision of the operated side or underwent surgery of the contralateral side was not eligible for evaluation. Besides, previous studies stated that the periprosthetic remodeling stabilizes in the second year, and only marginal changes of BMD occurred after the 12th postoperative month [19, 20]; therefore patient who had undergone primary unilateral THA within one year would also be excluded.

### 2.2. Implant Features

**Figure 1:**
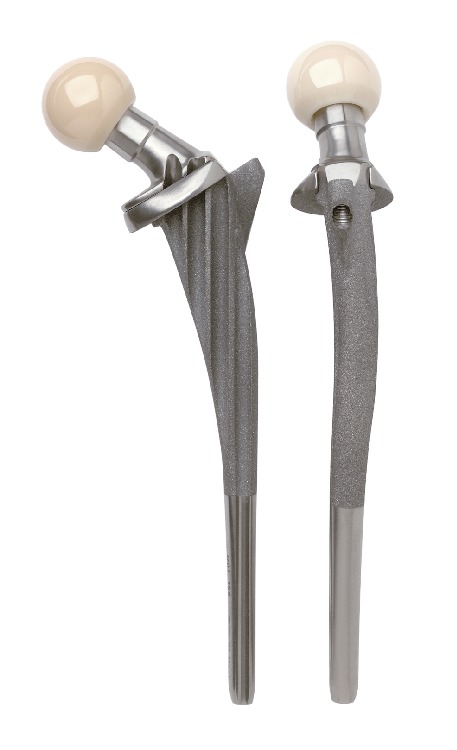
The characteristics of the Ribbed hip system.

**Figure 2:**
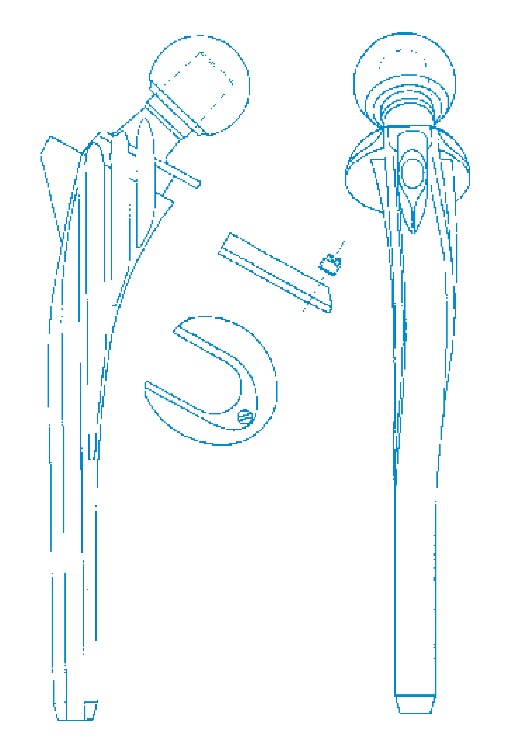
Illustration of the detachable collar of the Ribbed hip system.

**Figure 3:**
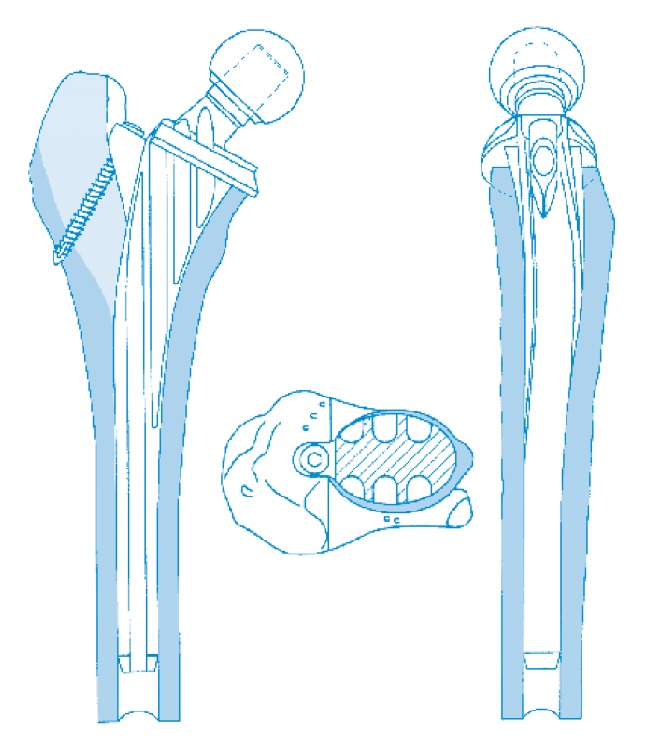
Illustration of the deep grooves and reduced cross section of the Ribbed hip system.

The Ribbed stem was designed with the following characteristics (see Figure 1) [21]: (i) the proximal stem portions and the underside of the detachable collar are covered with a microporous calcium phosphate coatings [22]; (ii) the prosthesis is anatomically S-shaped as the anatomical curvature of the femoral medullary canal, which was designed to realize insertion of the maximum allowable stem size and achieve better form closure and intended to reduce the rotational forces affecting the prosthetic anchorage [23]; (iii) a lateral fin at the proximal stem was designed aiming to enhances the primary stability against rotational forces after implantation [24]; (iv) furthermore, an anchoring screw through a bore hole in the lateral fin can be screwed into the greater trochanter intended to further enhance primary fixation, achieve better implant/bone composite, which was also designed aiming to reduce the compressive load onto the calcar during the initial postoperative stage [24]; (v) a prosthesis collar was designed aiming to reintroduce the physiological forces into the femur after the resection of the femoral neck, and the collar was designed to be detachable and can be removed to pack additional bone material into the grooves after the prosthesis stem has been positioned (see Figure 2); (vi) the stem was designed with deep grooves, which intended to reduce the cross section of the stem, increase the modulus of elasticity of the stem, and ultimately reduce the stress shielding or excessive stiffening of the proximal femur caused by the metallic implant (see Figure 3) [21, 25].

### 2.3. Bone Mineral Density

Dual-energy X-ray absorptiometry (DEXA) is the recognized standard method for clinical assessment of skeletal health, which is an accurate quantitative radiological procedure that can detect small changes in bone mass and reflect tiny change of BMD around the prosthesis [26–29]. According to the Gruen's method [30], BMD measurements were performed using the Discovery DEXA system and Hologic “metal-remove” software (Hologic, Inc., MA, US) in seven areas on both prosthetic side and contralateral side.

### 2.4. Radiographic Evaluation

Standardized anteroposterior and lateral plain radiography were performed for qualitative evaluation. The radiographic images were analyzed to evaluate the contact between the detachable collar and the medial calcar, the fixation of anchoring screw to the greater trochanter, the fitness of the distal stem within the isthmus of the femur, and the femoral stem alignment [31]. Furthermore, heterotopic ossification around the stem was graded according to Brooker classification [32]; radiolucent line around the stem was assessed according to the Gruen zones [30]; the type of fixation was graded according to the criteria of Engh [33]; and the D'Antonio method was referenced to evaluate whether the prosthesis was subsiding, shifting, or loosening [34]. Radiographs were put into a unique box, and identification of patient and temporal sequence of the images were blinded. Each radiographic image was evaluated by two researchers (orthopaedic fellows trained to X-ray image analysis) separately and then was recorded by mean of the two values. Moreover, postoperative functional outcomes were assessed using the Harris Hip Score (HHS), Western Ontario and McMaster Universities Arthritis Index (WOMAC) Score, and thigh pain.

### 2.5. Statistical Analysis

The sample size was calculated based on data from our preliminary results of this study, which detected a mean (standard deviation) BMD change of 0.1 (0.19) g/cm^2^ between the prosthetic and contralateral side. With these assumptions, we estimated that 38 participants would provide 90% power to detect a significant difference (5% type I error and 10% type II error,* P*=0.05, two-sided). To compensate for any nonevaluable patients, the authors planned to enroll 10% more patients.

All measurement data were expressed as mean ± standard deviation (SD) or median (range). The counting data was represented by the ratio. Differences between the prosthetic side and the contralateral side were evaluated by a paired* t*-test when normality assumptions are satisfied, otherwise the equivalent nonparametric test would be used. When analyzing paired data, we would first calculate the difference or percentage between two measurements in the same subject. To further explore confounders that might influence bone remodeling around prosthesis, subgroup analyses were carried out on the potential factors including primary disease and body mass index (BMI).* P*<0.05 was determined as statistically significant. All statistical analysis was performed using statistical software SPSS, version 21.0 (SPSS Inc., Chicago, IL).

## 3. Results

### 3.1. Patients' Characteristics

A total of 41 patients met the criteria and were included in the final analysis (see Table 1), including 19 males and 22 females, with a mean age of 62.07 years (range, 33~79 years) and average BMI of 24.65±3.33 kg/m^2^.

Postoperative follow-up appointment is completed and ranges from 14 to 119 months with a mean follow-up of 34.02±17.47 months. The disease spectrum consisted of avascular necrosis (AVN) (16 cases), developmental dysplasia of the hip (12 cases), femoral neck fractures (9 cases), and hip osteoarthritis (4 cases). The mean HHS score was 93.13 points (range: 81.00~98.00), the mean WOMAC score was 5.20 points (range: 0~23.00), and three patients described intermittent mild thigh pain at the final follow-up. The hip function of the prosthetic side was obviously improved, and functional evaluation demonstrated that most of patients were satisfied in the postoperative follow-up.

### 3.2. Comparison of BMD

The postoperative BMD changes in the Gruen zones of the periprosthetic bone are shown in Table 2.

**Figure 4:**
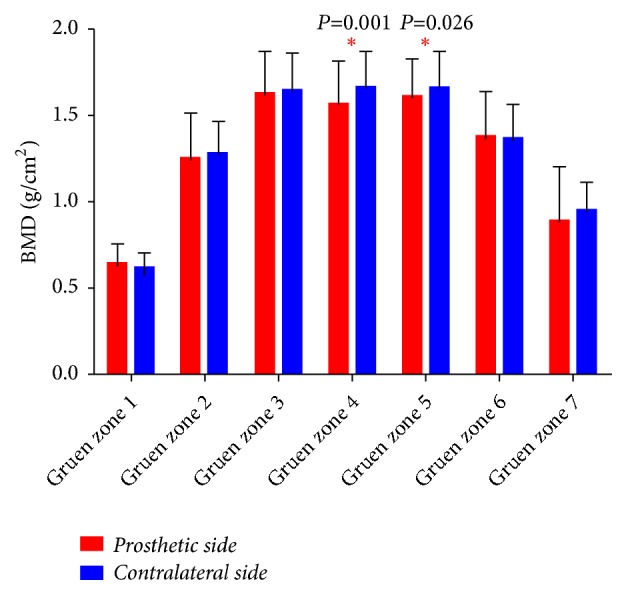
The comparison of periprosthetic bone mineral density changes between the prosthetic side and the contralateral side.

Compared with the contralateral unoperated side, significant reductions of periprosthetic BMD were detected in the distal zone (Gruen zone 4 BMD: 1.665±0.198* versus* 1.568±0.242 g/cm^2^,* P*=0.001) and middle distal zone (Gruen zones 5 BMD: 1.660±0.209* versus* 1.608±0.215 g/cm^2^,* P*=0.026) on the prosthetic side. No significant differences of periprosthetic BMD changes were detected in prosthetic side compared to the contralateral side, although marginal BMD changes were detected (see Figure 4).

The results of subgroup analyses are presented in Table 3, which indicated no significant correlation between periprosthetic BMD changes and primary disease or BMI. Notably, BMD was consistently lower in almost every zone both in operated and unoperated sides in patients diagnosed with AVN, but did not reach statistical significance.

### 3.3. Radiological Evaluation

The radiographic evaluation and analyses of the femoral component are shown in Table 4. Most cases were classified as good or fair contact between the prosthesis and the femur; 38 hips (92.68%) presented neutral femoral stem alignment; three mild varus femoral stem alignments were registered but did not demonstrate any signs of loosening. Ten patients developed grade I heterotopic ossification and one patient developed grade II heterotopic ossification; 28 patients presented visible spot welds and 6 patients with incomplete pedestal indicated bone ingrowth and solid fixation of the femoral stem. All of the patients had radiographically stable femoral component and no radiolucent lines were detected. There was no case of prosthetic subsidence, migration, or loosening in X-ray image for any reason.

## 4. Discussion

### 4.1. Main Findings

To the best of our knowledge, this is the first study that applied DEXA to explore the periprosthetic BMD changes and bone remodeling pattern in patients who underwent primary unilateral THA with the Ribbed anatomic stem. No statistically significant periprosthetic BMD change was detected in the proximal femur compared with the contralateral side, while significant periprosthetic bone loss was detected in the distal and middle distal zones (Gruen zones 4 and 5). Additionally, X-ray indicated solid fixation of the femoral stem and detected no preliminary sign of loosening.

### 4.2. Comparison with Previous Studies

Plenty of studies have followed-up the postoperative clinical outcomes of the Ribbed stem in many clinical centers (see Table 5) [35–43]. However, these studies mainly used radiography and functional scores as evaluation criteria, demonstrated excellent clinical results with stable bone ingrowth fixation, and suggested desirable postoperative functional scores. In comparison, we further adopted DEXA technique to quantitative evaluate the periprosthetic BMD changes and found notable declines of BMD in the distal and middle distal regions (Gruen zones 4 and 5), while BMD in other Gruen zones were marginally changed or virtually comparable with the contralateral unoperated side.

### 4.3. Implant-Related Factors

**Figure 5:**
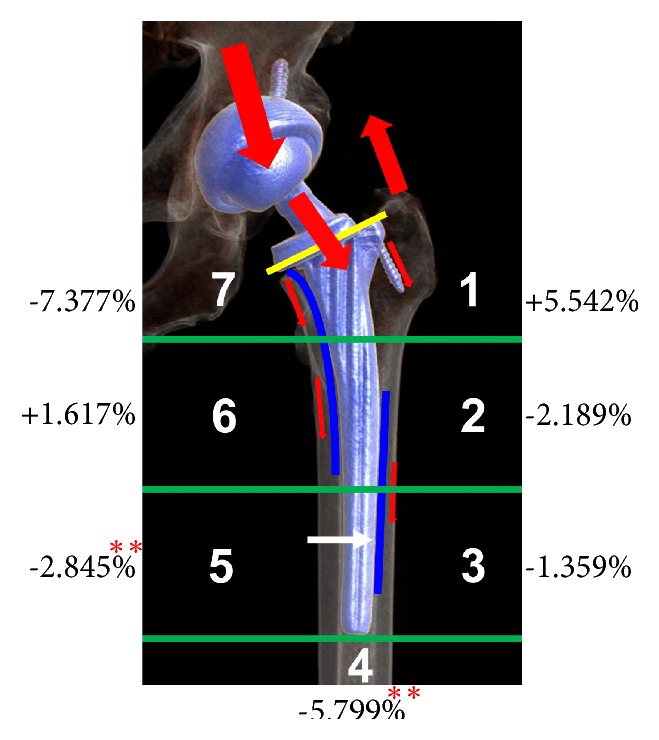
The stress transmission of the proximal femur with the Ribbed stem.

The possible mechanism of periprosthetic bone remodeling and BMD changes is nonphysiological strain distribution and inevitable stress shielding [44, 45], although the Ribbed stem is a type of anatomical prosthesis designed to achieve proximal femoral fixation and reintroduce normal physiological stress conduction, which can transfer more compression forces to the metaphyseal cortical bone and stimulate physiological forces into the femur. The changes of BMD imply that more proximal stress is distributed in proximal lateral region (Gruen zone 1) and middle medial zone (Gruen zone 6), accompanied with evident stress shielding in distal zone (Gruen zone 4) and medial distal zone (Gruen zone 5) (see Figure 5).

Remarkably, previous studies have reported that Gruen zones 1 and 7 are two of the most serious osteolysis areas due to stress shielding [46]. However, for the Ribbed stem with anchoring screw in Gruen zones 1 and collar in Gruen zones 7, bone resorption in these two areas was not statistically significant when compared with the contralateral side, which indicated that the anchoring screw and prosthesis collar might transfer more physiological bone loading, strengthen proximal local contact stresses, and reduce stress shielding in Gruen zones 1 and 7, and proximal load transmission approaches physiological patterns. This does not take away from the fact that Gruen zone 7 observed high SDs, which indicates a large range of BMD values and high diversity of periprosthetic BMD changes in Gruen zone 7. This variability might attribute to the contact and physiological loading between the detachable collar and the medial calcar.

During normal walking, the proximal femur is subjected to compression loads on the medial side and tension stress on the lateral side [47]. The kinematic femur is naturally more compliant than a solid, canal-filling metal prosthesis. Theoretically, physiological loading generates compression loading and interface shear stresses [48], which separately concentrated on Gruen zones 6 and 3, and bone adaptation to the implant of both areas would secure the interface. Consequently, physiological loading unnaturally focused on contact areas with the femoral component, while stress on distal and medial distal areas (Gruen zones 4 and 5) were shielded (see Figure 5) [49]. Therefore, local contact stresses and stress shielding as well as shear stresses play important roles in femoral remodeling of cementless prostheses [50].

During the past decades, the concepts for designing and fabricating implants have been dramatically improved. Initially, hip implants were designed to impact into the distal part of the femur to increase anchoring area and achieve optimum primary stability. Straight stems were then designed, which transfers most of the physiological loads into the distal femur, and the proximal femur becomes less dense and weaker under the stress shielding effect [51–53]. Thus, anatomic stems are invented to overcome the shortcomings, which are fixed in the upper part of the femur for more physiological bone loading to reduce the negative effects of nonphysiological strain distribution and stress shielding [54, 55]. Previous studies compared anatomic stem versus straight stem by using DEXA which implied that the extensive proximal, more physiological bone loading of the anatomic stem led to better preservation of the proximal femur [20, 56, 57].

However, BMD loss in the medial femoral neck (calcar) and greater trochanter (especially Gruen zones 7 and 1) cannot be avoided despite extensive proximal anchoring of the anatomic stem [20, 52, 56, 57]. Thus, optimization of implant design to simulate the physiological load transfer would be perfect to avoid periprosthetic bone resorption of the proximal femur. Consequently, short hip stems and even neck stems have become the latest concepts. These stems help preserve the femoral neck, keep the anatomical elasticity of the femur and introduce the forces in the upper part of the femur [18, 51, 58–64]. And studies evaluated periprosthetic bone remodeling with DEXA showed a more balanced remodeling and favorable load transfer of the short stem in comparison to standard hip stem [16, 51, 60–65].

The implant design and concept development are returning to origin surgical intervention, hip resurfacing, which is unlikely to result in stress shielding of the proximal femur [65]. However, stem shortening would reduce stress shielding as well as the initial stability, and trade-off between stress shielding and initial stability is necessary when designing new cementless stems [66]. In addition, fabrication and surface modification techniques have been developed to promote osteointegration, enhance tribological performance, and prolong the life of implants [67, 68]. These techniques could also have significant influence on the periprosthetic BMD changes [69–71].

### 4.4. Nonimplant-Related Factors

Many nonimplant-related factors including age, sex, BMI, primary disease, bone quality, osteoporosis treatment, and daily activity might have potential impact on periprosthetic bone remodeling [72–74], although subgroup analyses indicated no correlation between periprosthetic BMD change and primary disease or BMI. Noticeably, bone remodeling around prosthesis in the patients with AVN will be permanently influenced by primary disease [75], the difference might be gradually increased in long-term follow-up, and antiosteoporosis therapy should be highly recommended in this population.

### 4.5. Future Perspectives

With the rapid development of materials science and three-dimensional printing technology in recent years, patient-specific hip implants will probably be the best available implant solution [76]. Previous studies have shown that individualized, customized femoral prostheses have more advantages in the distribution of stress around the components and bone osteointegration [54–56, 77]. Furthermore, systematic review of current implants design and following periprosthetic bone remodeling will improve the design of patient-specific hip implants [69, 78].

### 4.6. Limitations

Our study has several limitations. First, this is a cross-sectional follow-up study rather than a prospective consecutive study, and preoperative baseline data or continuous follow-up data was not available as control arms. Therefore, we compared the BMD changes in the prosthetic side with the contralateral side, since the method is self-controlled, and estimation within individuals rather than separate controls would help to control potential influential factors (e.g., age, sex, primary disease, osteoporosis treatment, and daily activity) and cancelled out time variant factors (e.g., BMI; bone quality); it would be reasonable to take the unoperated side as a reference. Second, although DEXA is the preferred diagnostic tool for measuring periprosthetic BMD, the method of Gruen zones has inherent limitation, which divided the proximal femur into medial and lateral parts for evaluation and is unable to evaluate the anterior and posterior areas. Quantitative computer-tomography assisted osteodensitometry could be a better method [17, 79]. Third, our study included relatively small number of patients with short to midterm follow-up, which limits the strength of the conclusions. Finally, although we applied subgroup analyses to evaluate factors that might affect periprosthetic bone remodeling, the sample size is inadequate to discuss subgroup analysis and clinical outcomes; thereby correlation between BMD and these factors should be interpreted with caution and further investigation is warranted.

## 5. Conclusion

In summary, in patients who had undergone THA with the Ribbed femoral stem, the periprosthetic BMD was well maintained in the proximal femur, while BMD was reduced in the distal and medial distal femur around the stem due to stress shielding. The proximal features of Ribbed stem are well worth referencing in designing of novel hip implants. Further clinical investigations are required to examine the good results, particularly in terms of long-term survival. And explorations regard implant-related and nonimplant-related factors that influence osteointegration and periprosthetic BMD changes are warrant.

## Figures and Tables

**Table 1:** Characteristics and clinical data of patients included in this study.

Male/Female	19/22
Age (years)	62.07 ± 10.85^†^
Height (cm)	161.38 ± 7.01^†^
Weight (kg)	64.30 ± 9.98^†^
BMI (kg/m^2^)	24.65 ± 3.33^†^
Postoperative follow-up (month)	34.02 ± 17.47;^†^ 34 (14, 119)^‡^
Primary disease (n)	
AVN	16
DDH	12
FNF	9
Osteoarthritis	4
Calcium supplements (n)	20
Harris hip score	95.00 (81.00, 98.00)^ ‡^
WOMAC score	4.00 (0, 23.00)^ ‡^
Thigh pain	3 cases with mild intermittent thigh pain

AVN: avascular necrosis; DDH, developmental dysplasia of the hip; FNF, femoral neck fracture.

^†^Values are expressed as mean and standard deviation.

^‡^Values are expressed as median and range.

**Table 2:** Periprosthetic BMD changes between operated and unoperated contralateral hip of the 7 Gruen zones.

Gruen zone	Prosthetic side BMD (g/cm^2^)	Contralateral side BMD (g/cm^2^)	Mean change	BMD percentage %	BMD change %	*P*-value
Gruen zone 1	0.640 ± 0.116	0.612 ± 0.087	0.028	105.542 ± 19.716	5.542	0.131
Gruen zone 2	1.249 ± 0.258	1.280 ± 0.181	-0.031	97.811 ± 17.211	-2.189	0.361
Gruen zone 3	1.624 ± 0.244	1.648 ± 0.209	-0.024	98.641 ± 10.059	-1.359	0.317
Gruen zone 4	1.568 ± 0.242	1.665 ± 0.198	-0.097	94.201 ± 10.231	-5.799	0.001
Gruen zone 5	1.608 ± 0.215	1.660 ± 0.209	-0.052	97.155 ± 8.448	-2.845	0.026
Gruen zone 6	1.374 ± 0.261	1.362 ± 0.201	0.012	101.617 ± 17.810	1.617	0.730
Gruen zone 7	0.888 ± 0.312	0.952 ± 0.157	-0.064	92.630 ± 27.005	-7.377	0.119

BMD, bone mineral density. Values are mean (SD) or percentage (%) (n=41).

**Table 3:** Subgroup analysis of the potential influential factors of bone mineral density.

	Prosthetic side BMD (g/cm^2^)			Contralateral side BMD (g/cm^2^)		

Gruen zone	Non-AVN (n=25)	AVN (n=16)	*P*-value	Non-AVN (n=25)	AVN (n=16)	*P*-value

Gruen zone 1	0.646 ± 0.106	0.631 ± 0.132	0.681	0.614 ± 0.091	0.610 ± 0.084	0.886
Gruen zone 2	1.275 ± 0.238	1.208 ± 0.288	0.422	1.267 ± 0.165	1.300 ± 0.208	0.572
Gruen zone 3	1.653 ± 0.221	1.579 ± 0.279	0.350	1.652 ± 0.206	1.643 ± 0.220	0.895
Gruen zone 4	1.605 ± 0.220	1.509 ± 0.270	0.222	1.671 ± 0.184	1.655 ± 0.225	0.798
Gruen zone 5	1.618 ± 0.221	1.594 ± 0.211	0.729	1.679 ± 0.172	1.630 ± 0.260	0.470
Gruen zone 6	1.407 ± 0.238	1.323 ± 0.295	0.321	1.377 ± 0.167	1.338 ± 0.248	0.585
Gruen zone 7	0.915 ± 0.301	0.847 ± 0.334	0.504	0.984 ± 0.155	0.903 ± 0.152	0.104

Gruen zone	BMI ≤24 (n=19)	BMI>24 (n=22)	*P*-value	BMI ≤24 (n=19)	BMI>24 (n=22)	*P*-value

Gruen zone 1	0.631 ± 0.106	0.647 ± 0.125	0.680	0.603 ± 0.086	0.620 ± 0.089	0.530
Gruen zone 2	1.228 ± 0.291	1.267 ± 0.230	0.632	1.221 ± 0.171	1.331 ± 0.178	0.051
Gruen zone 3	1.552 ± 0.292	1.686 ± 0.179	0.081	1.613 ± 0.209	1.678 ± 0.209	0.327
Gruen zone 4	1.504 ± 0.263	1.623 ± 0.213	0.118	1.625 ± 0.191	1.699 ± 0.202	0.232
Gruen zone 5	1.531 ± 0.234	1.675 ± 0.176	0.031	1.605 ± 0.222	1.706 ± 0.189	0.123
Gruen zone 6	1.332 ± 0.266	1.411 ± 0.258	0.342	1.349 ± 0.205	1.373 ± 0.201	0.699
Gruen zone 7	0.920 ± 0.354	0.861 ± 0.275	0.554	0.943 ± 0.191	0.961 ± 0.125	0.717

BMD, bone mineral density; AVN: avascular necrosis; BMI, body mass index.

**Table 4:** Radiographic evaluation of the femoral component.

Evaluation content	No. of patients	Percentage (%)
Contact collar-calcar		
Good	25	60.97
Fair	12	29.27
Poor	4	9.76
Fixation screw-trochanter		
Good	21	51.22
Fair	14	34.15
Poor	6	14.63
Contact prosthesis-isthmus		
Good	37	90.24
Fair	4	9.76
Poor	0	0
Femoral stem alignment		
Varus	3	7.32
Neutral	38	92.68
Valgus	0	0
Heterotopic ossification		
I	10	24.39
II	1	2.44
III	0	0
IV	0	0
Pedestal sign		
Incomplete	6	14.63
Complete	0	0
Spot welds	28	68.29
Reactive lines (<2 mm)	14	34.15
Radiolucent line (>2 mm)	0	0
Femoral stem subsidence, migration or loosening	0	0

**Table 5:** Comparison with other previous studies.

Study	Publication year	Duration of follow-up	No. of cases	Hip Harris score (≥90)	VAS score	Radiolucent lines in Gruen zones							Engh/Bobyn method	D' Antomo method
						Gruen zone 1	Gruen zone 2	Gruen zone 3	Gruen zone 4	Gruen zone 5	Gruen zone 6	Gruen zone 7		
Sweetnam et al. [35]	1995	72-93 months	12	−	−	−	−	−	−	−	−	−	−	7 cases prosthetic loosening
Zhou et al. [36]	2003	6-54 months	87	95.30%	−	2 cases	1 case	−	−	−	−	1 case	All with good osseointegration	−
Wang et al. [37]	2006	6-61 months	163	94.50%	−	2 cases		−	−	−	−	1 case	−	−
Lei et al. [38]	2007	8-61 months	38	93.50%	4 cases with mild thigh pain	−	−	−	−	−	−	−	All with good osseointegration	−
Liu et al. [39]	2008	2-10.5 months	1346	96.30%	−	2 cases	−	−	−	−	−	1 case	All with good osseointegration	−
Mao et al. [40]	2010	0.5-8 years	84	54.76%	3 cases with mild thigh pain	−	−	3 cases	−	3 cases	−	−	−	4 cases prosthetic subsidence
Mao et al. [41]	2010	9-12 years	26	69.23%	1 case with mild thigh pain	−	1 case	1 case	−	1 case	1 case	−	−	−
Shi et al. [42]	2012	3-14 years	662	95.70%		1 case	−	−	−	−	−	1 case	All with good osseointegration	
Jiang et al. [43]	2014	2-4 years	52	−	2 cases with mild thigh pain,1 case with medium thigh pain	5 cases	1 case	2 cases	−	−	−	7 cases	All with good osseointegration	2 cases prosthetic varus, 4 cases prosthetic vagus

## Data Availability

The data used to support the findings of this study are available from the corresponding author upon request.

## Abbreviations

AVN: Avascular necrosis
BMD: Bone mineral density
BMI: Body mass index
DDH: Developmental dysplasia of the hip
DEXA: Dual-energy X-ray absorptiometry
FNF: Femoral neck fracture
HA: Hydroxyapatite
HHS: Harris Hip Score
SD: Standard deviation
THA: Total hip arthroplasty
VAS: Visual Analogue Scale
WOMAC: Western Ontario and McMaster Universities Arthritis Index.
